# CEA dynamics for predicting response after anti-EGFR monoclonal antibody treatment in metastatic colorectal cancer

**DOI:** 10.1038/s41598-023-33811-x

**Published:** 2023-04-25

**Authors:** Sora Kang, Sun Young Kim, Yong Sang Hong, Tae Won Kim, Ki Eun Choi, Min Jung Kim, Jeong Eun Kim

**Affiliations:** 1 Department of Medical Oncology, Asan Medical Center, University of Ulsan College of Medicine, 88, Olympic-ro 43-gil, Songpa-gu, Seoul, 05505 South Korea; 2 Division of Hemato-Oncology, Department of Internal Medicine, Chungnam National University Hospital, Daejeon, South Korea

**Keywords:** Cancer, Colon cancer, Rectal cancer

## Abstract

Carcinoembryonic antigen (CEA) is the most widely used tumor marker in metastatic colorectal cancer (mCRC). However, its potential as a predictive marker of progression in mCRC during systemic chemotherapy, particularly in patients receiving monoclonal antibodies as a combination therapy, has remained of interest. Herein, we investigated whether CEA changes could predict disease progression and clinical outcomes in patients with mCRC cotreated with systemic chemotherapy and/or biologic agents. A total of 1261 patients with mCRC undergoing a first-line systemic treatment were included in this retrospective study. We analyzed the optimal cut-off value for CEA changes to predict progression at the first response evaluation by the treatment arm (chemotherapy alone, chemotherapy plus anti-vascular endothelial growth factor (VEGF) monoclonal antibody [mAb], and chemotherapy plus anti-epidermal growth factor receptor [EGFR] mAb). These cut-off values were then used to predict overall survival (OS) and progression-free survival (PFS). When stratified by their treatment arm, 891 (70.6%), 266 (21.0%), and 104 (8.2%) of the study patients were included in the chemotherapy alone-, anti-VEGF mAb, and anti-EGFR mAb groups, respectively. The optimal CEA cut-off values were 16.5% and 38.9% increase in the whole cohort and anti-EGFR mAb group, respectively, and these values showed high sensitivity and specificity for predicting disease progression. The patients in the entire population and anti-EGFR mAb group with CEA changes below these cut-off values showed significantly better OS and PFS outcomes compared those whose changes were above cut-off values. Among the patients with mCRC treated with anti-VEGF mAb, no associations were found between OS or PFS outcomes and CEA changes. CEA is potentially a good surrogate marker for predicting disease progression and survival outcomes in patients with mCRC receiving first-line systemic chemotherapy alone or chemotherapy with anti-EGFR mAb, whereas it is less effective in those treated with anti-VEGF mAb.

## Introduction

Although novel biomarkers such as circulating tumor DNA (ctDNA) are currently under investigation^1^, serum carcinoembryonic antigen (CEA) is still the most widely used tumor marker for colorectal cancer (CRC) worldwide^2,3^. CEA has a unique advantage compared with ctDNA in terms of its inexpensive cost and reduced time required to wait for results, and it is the marker of choice for postoperative surveillance and monitoring disease response during systemic chemotherapy in CRC^2–5^.

In real-world clinical practice, it is important for medical oncologists to define whether the cancer is progressive or not during systemic chemotherapy for optimizing the treatment. Since changes in the CEA level may reflect tumor burden^6,7^, several previous studies of CRC have reported a correlation between changes in CEA level and disease response, suggesting that changes in CEA level may be a good surrogate marker for predicting disease response during systemic chemotherapy^8–14^. However, most of those studies had a limited sample size, and most of the included patients were treated with chemotherapy alone.

Currently, anti-vascular endothelial growth factor (VEGF) monoclonal antibody (mAb) and anti-epidermal growth factor receptor (EGFR) mAb are recommended by both the National Comprehensive Cancer Network (NCCN) and European Society of Medical Oncology (ESMO) guidelines as part of the first-line systemic treatments in combination with cytotoxic chemotherapy for eligible patients with CRC^15,16^. As the chemotherapy and mAb combination was established as the standard-of-care for eligible patients, the clinical value of CEA changes in predicting disease response in patients with metastatic CRC (mCRC) treated with targeted therapy remains of interest.

In this study, we aimed to evaluate the association between different levels of CEA changes and disease response at the time of first response evaluation in patients with mCRC treated with first-line chemotherapy, especially those treated with an mAb combination. We divided patients with mCRC in our cohort into three groups and analyzed the changes in CEA and assessed the serum CEA threshold that could be used to predict disease response by treatment regimen i.e., chemotherapy alone, chemotherapy with anti-VEGF mAb, and chemotherapy with anti-EGFR mAb. In addition, we evaluated the prognostic value of these cut-off values for predicting long-term clinical outcomes in our cohort of patients with mCRC.

## Method

### Patients

We identified patients treated for mCRC between 2008 and 2015 (n = 2962) at Asan Medical Center, a tertiary hospital in Seoul, South Korea. Among this initial population, we excluded patients for whom CEA data at baseline or at their first response evaluation were not available (n = 766) as well as those whose disease response information was not evaluable due to follow-up loss, death, and/or an unavailable restaging image (n = 935). For inclusion in the analyses, a baseline CT must have been performed within 4 weeks of chemotherapy initiation, and a follow-up CT scan for the response evaluation must have been performed within 12 weeks of chemotherapy initiation. Moreover, the CEA level must have been measured within 2 weeks of the response evaluation scan. A final total of 1261 patients were included in the analysis (Supplementary Fig. S1). We retrospectively reviewed their medical records and collected data on age, sex, tumor characteristics including the grade and mutation, mismatch repair (MMR) and microsatellite instability (MSI) status, BRAF mutation status, CEA levels at baseline and at the time of the first response evaluation, treatment regimen, and response assessment. The systemic disease response was evaluated using the Response Evaluation Criteria In Solid Tumors (RECIST) version 1.1. Enzyme immunoassays (ELISA-2-CEA kit; CIS Biointernational, Marcoule, France) were used to measure the serum CEA.

This study was conducted in accordance with the Declaration of Helsinki and was reviewed and approved by the Institutional Review Board (IRB) of Asan Medical Center (Approval date: Sep 14, 2017; IRB No.: 2017-1098). The requirement for patient informed consent was waived by the IRB of Asan Medical Center due to the retrospective nature of the study.

### Outcomes

A central goal of these analyses was to determine whether CEA changes from the period before chemotherapy initiation to the first response evaluation could predict CRC progression on the first response evaluation imaging scan, which was conducted 6–8 weeks after initiation of chemotherapy or when disease progression was clinically suspected. We aimed to define an optimal cut-off value for the serum CEA level at the time of the first response evaluation imaging scan to predict the treatment response (progressive disease [PD] vs non-PD) in patients with mCRC undergoing first-line chemotherapy. In addition, we aimed to evaluate the association between any CEA changes at the first response evaluation and clinical outcomes, such as progression-free survival (PFS) and overall survival (OS) among patients with mCRC.

### Statistical analysis

We conducted our present analysis according to the treatment arm i.e. chemotherapy alone, chemotherapy plus anti-VEGF mAb, and chemotherapy plus anti-EGFR mAb. The optimal cut-off values for the serum CEA changes were estimated using receiver operating characteristic (ROC) curve analysis. Using these cut-off values, the sensitivity, specificity, and positive and negative predictive values were estimated to predict their performance. A multivariable logistic regression model including age (age ≥ 60 years vs < 60 years), tumor location (right vs left), percentage change in CEA level (above vs below the cut-off value), and BRAF mutation status (wild type vs mutant) was used to estimate the adjusted odds ratio (OR) and corresponding 95% confidence interval (CI). Patients for whom information on the tumor location and BRAF mutation status were not available were excluded in this model; thus, a total of 1109 patients were included.

The Wilcoxon rank-sum test was used to compare the percentage change in the CEA level between patients confirmed to have progressive or non-progressive disease at the first response evaluation. To analyze categorical variables, the Chi-square test or Fisher’s exact test was used as appropriate. The OS was calculated from the date of the first-line chemotherapy initiation to the date of death from any cause. The PFS was calculated from the date of first-line chemotherapy initiation to the date of disease progression or death, whichever occurred first. The Kaplan–Meier method was used to estimate the OS and PFS, and the log-rank test was used to compare the clinical outcomes of the subgroups. Multivariable Cox proportional-hazards models were used to estimate the hazard ratio [HR] and 95% CIs for evaluating the prognostic value of each variable for OS and PFS. P values < 0.05 were considered to indicate statistical significance, and all analyses were conducted using statistical software R (Version 4.0.5, Vienna, Austria).

## Results

### Baseline characteristics

The baseline characteristics of the included patients are summarized in Table 1. The median age was 57 years (range, 20–82 years), and 61% (n = 775) of these patients were male. Most of the tumors in this population were located on the left side (n = 959, 76%), and 79% of the patients (n = 998) had a moderately differentiated tumor grade. When stratified using the treatment arm, 891 (70.6%), 266 (21.0%), and 104 (8.2%) patients were treated with chemotherapy alone, chemotherapy plus anti-VEGF mAb, and chemotherapy plus anti-EGFR mAb, respectively. The median baseline CEA was 8 ng/mL (interquartile range [IQR], 2–57 ng/mL), and the median CEA at the first response evaluation was 5 ng/mL (IQR, 2–34 ng/mL). The median duration from first-line chemotherapy initiation to the first response evaluation was 7.57 weeks (range, 1.57–12 weeks). The most commonly used chemotherapy regimens among our current study patients were folinic acid, fluorouracil, and irinotecan (FOLFIRI) in the chemotherapy alone group (n = 354, 40% of 891), bevacizumab plus FOLFIRI (n = 181, 68% of 266) in the chemotherapy plus anti-VEGF mAb group, and cetuximab plus FOLFIRI (n = 86, 83% of 104) in the chemotherapy plus anti-EGFR mAb group (Supplementary Table S1). None of the patients were treated with panitumumab. The number of patients who showed progressive disease at the first response evaluation was 142 (16%), 18 (6.8%), and 8 (7.7%) in the chemotherapy alone, chemotherapy plus anti-VEGF mAb, and chemotherapy plus anti-EGFR mAb groups, respectively.

**Table 1:** Baseline characteristics of the included patients.

Variables	All patients (n = 1261)	CTx alone (n = 891)	CTx plus Anti-VEGF mAb (n = 266)	CTx plus anti-EGFR mAb (n = 104)	P-value
Age, years Median (range)	57 (20–82)	57 (20–82)	56 (27–79)	54 (25–77)	0.016
Sex					0.3
Male	775 (61%)	555 (62%)	153 (58%)	67 (64%)	
Female	486 (39%)	336 (38%)	113 (42%)	37 (36%)	
Primary tumor location					0.038
Right	296 (23%)	202 (23%)	78 (29%)	16 (15%)	
Left	959 (76%)	685 (77%)	186 (70%)	88 (85%)	
Multifocal	2 (0.2%)	2 (0.2%)	0 (0%)	0 (0%)	
Unknown	4 (0.3%)	2 (0.2%)	2 (0.8%)	0 (0%)	
Tumor grade					0.5
Well differentiated	91 (7.2%)	69 (7.7%)	13 (4.9%)	9 (8.7%)	
Moderately differentiated	998 (79%)	701 (79%)	213 (80%)	84 (81%)	
Poorly differentiated	117 (9.3%)	79 (8.9%)	29 (11%)	9 (8.7%)	
Unknown	55 (4.4%)	42 (4.7%)	11 (4.1%)	2 (1.9%)	
Baseline CEA, ng/mL Median (IQR)	8 (2–57)	7 (2–44)	11 (2–74)	17 (5–170)	< 0.001
CEA at first response evaluation, ng/mL, median (IQR)	5 (2–34)	5 (2–28)	6 (2–44)	7 (3–40)	0.2
Progression at first response evaluation	168 (13%)	142 (16%)	18 (6.8%)	8 (7.7%)	< 0.001
KRAS mutation					< 0.001
Wild type	716 (57%)	500 (56%)	118 (44%)	98 (94%)	
Mutant	436 (35%)	297 (33%)	139 (52%)	0 (0%)	
Unknown	109 (8.6%)	94 (11%)	9 (3.4%)	6 (5.8%)	
NRAS mutation					0.07
Wild type	326 (26%)	97 (11%)	174 (65%)	55 (53%)	
Mutant	15 (1.2%)	8 (0.9%)	7 (2.6%)	0 (0%)	
Unknown	920 (73%)	786 (88%)	85 (32%)	49 (47%)	
BRAF mutation					0.3
Wild type	1,062 (84%)	733 (82%)	236 (89%)	93 (89%)	
Mutant	52 (4.1%)	34 (3.8%)	16 (6.0%)	2 (1.9%)	
Unknown	147 (12%)	124 (14%)	14 (5.3%)	9 (8.7%)	
MMR status					0.2
Proficient MMR	857 (68%)	593 (67%)	192 (72%)	72 (69%)	
Deficient MMR	37 (2.9%)	31 (3.5%)	4 (1.5%)	2 (1.9%)	
Unknown	367 (29%)	267 (30%)	70 (26%)	30 (29%)	
MSI					> 0.9
MSS and MSI low	806 (63.9%)	574 (64.4%)	174 (65.4%)	58 (55.7%)	
MSI high	18 (1.4%)	14 (1.6%)	3 (1.1%)	1 (1.0%)	
Unknown	437 (35%)	303 (34%)	89 (33%)	45 (43%)	

CTx, chemotherapy; mAb, monoclonal antibody; IQR, interquartile range; MMR, mismatch repair; MSI, microsatellite instability; MSS, microsatellite stable.

### Dynamics of CEA changes at the first response evaluation according to treatment arm

**Figure 1:**
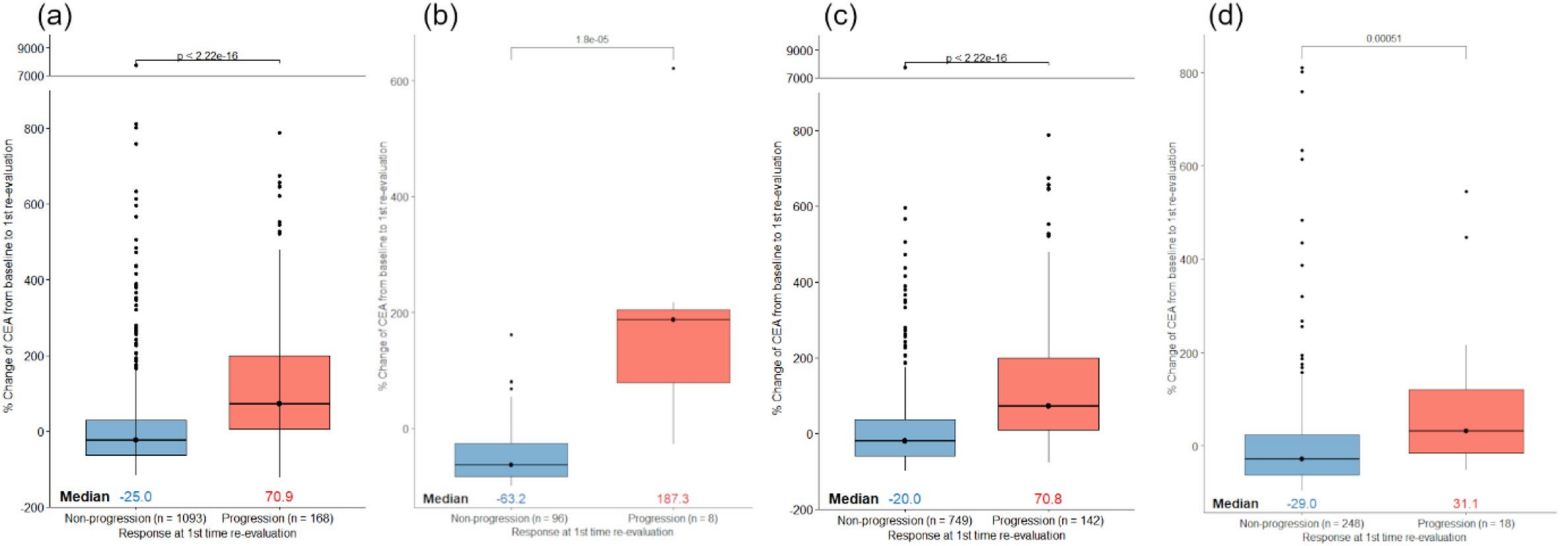
Boxplots of serum carcinoembryonic antigen (CEA) changes from baseline to the first response evaluation. (**a**) Whole cohort; (**b**) patients treated with chemotherapy plus anti-epidermal growth factor receptor (EGFR) monoclonal antibody (mAb); (**c**) patients treated with chemotherapy alone; (**d**) patients treated with chemotherapy plus anti-vascular endothelial growth factor (VEGF) mAb.

**Figure 2:**
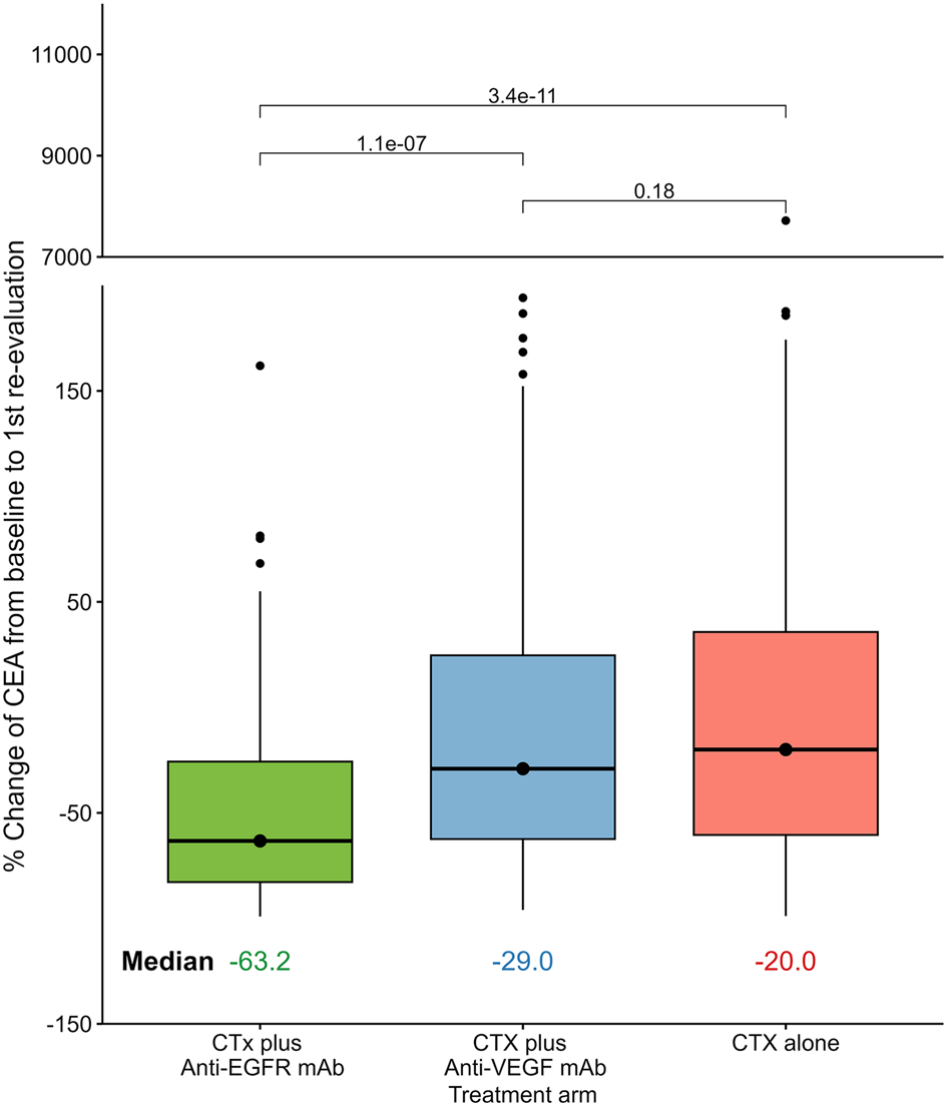
Boxplots of serum carcinoembryonic antigen (CEA) changes from baseline to the first response evaluation in patients without progressive disease (PD) according to treatment arm. CTX, chemotherapy; EGFR, epidermal growth factor receptor; VEGF, vascular endothelial growth factor.

In the whole cohort, the median percent change in CEA from baseline to the first response evaluation was − 25% in patients without PD and 70.9% in patients with PD (Fig. 1a, P < 0.001). In the groups based on the treatment arm, the median percent change in CEA from baseline to the first response evaluation in patients without and those with PD was − 63.2% and 187.3%, respectively, in the anti-EGFR mAb combination group (Fig. 1b, P < 0.001), − 20% and 70.8%, respectively, in the chemotherapy alone group (Fig. 1c, P < 0.001), and − 29.0% and 31.1%, respectively, in the anti-VEGF combination therapy group (Fig. 1d, P < 0.001). Among patients without PD, the median decrease in CEA was the largest in the anti-EGFR mAb treatment group (− 63.2%) compared with the chemotherapy alone (− 20%) and anti-VEGF mAb groups (− 29.0%; Fig. 2).

### Predicting the response to the first-line treatment at the first response evaluation using the CEA level

**Figure 3:**
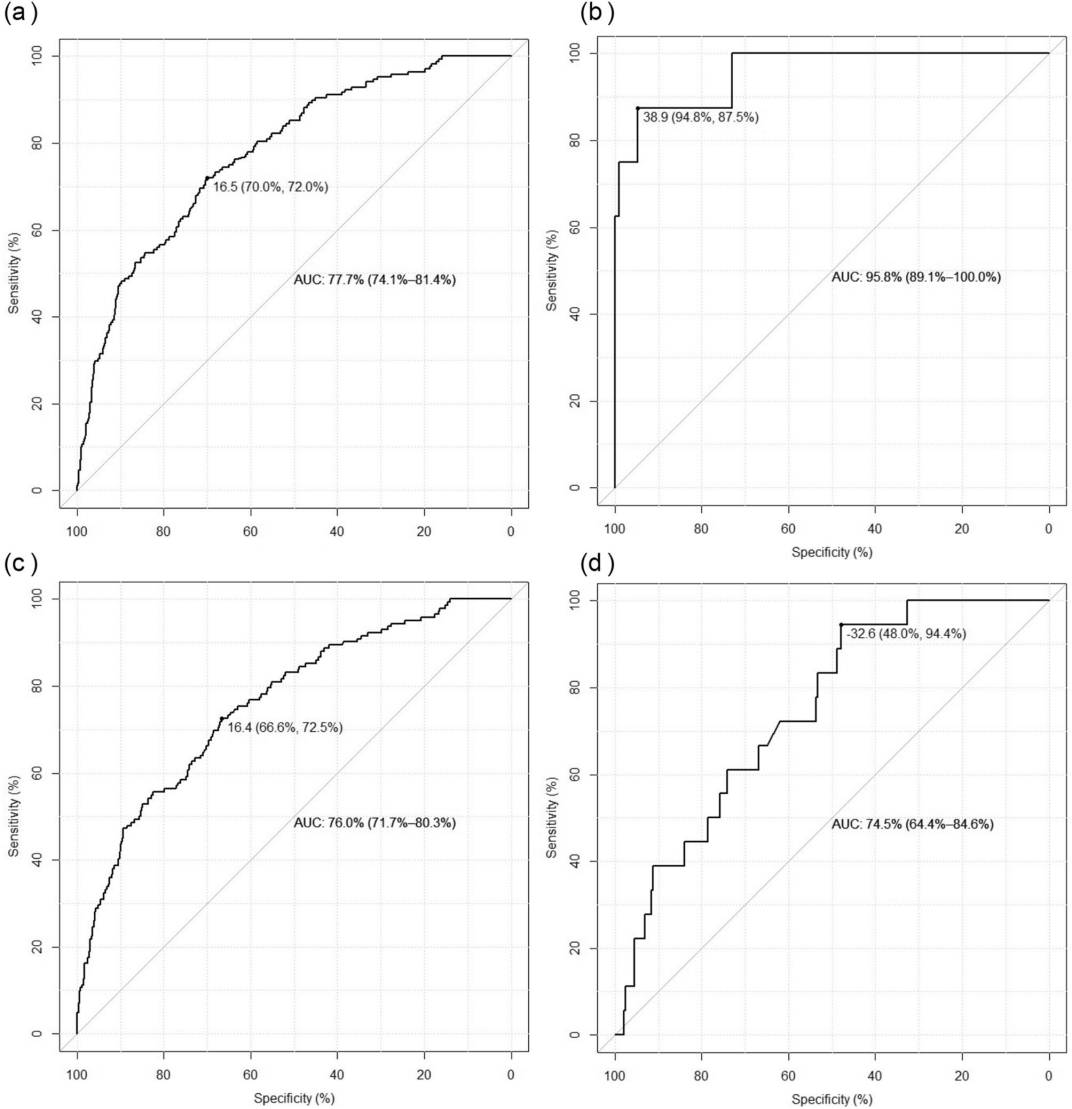
Receiver operating characteristic (ROC) curve analysis with the area under the curve (AUC) method in (**a**) whole cohort; (**b**) patients treated with chemotherapy plus anti-epidermal growth factor receptor (EGFR) monoclonal antibody (mAb); (**c**) patients treated with chemotherapy alone; (**d**) patients treated with chemotherapy plus anti-vascular endothelial growth factor (VEGF) mAb.

Using ROC curve analysis, the optimal cut-off value for the percent change in CEA to predict PD was defined for each treatment arm of our mCRC cohort. In the total population, this was estimated to be an increase of over 16.5% (area under the curve [AUC], 77%, 95% CI 74.1–81.4; Fig. 3a). The sensitivity and specificity of this cut-off value were 72.0% and 70.0%, respectively (Table 2). Patients with a CEA increase above 16.5% showed a higher probability of developing PD (adjusted OR; 4.06 [95% CI 2.80–5.90], P < 0.001; Table 3). Among the patients treated with the anti-EGFR mAb combination, the optimal cut-off value was defined as an increase of over 38.9% (AUC 95.8%; Fig. 3b). The sensitivity and specificity of this cut-off value for predicting PD and non-PD were 87.5% and 94.8%, respectively (Table 2). The CEA cut-off value using ROC curve analysis in the chemotherapy alone and anti-VEGF mAb groups is shown in Fig. 3c,d, respectively, and their sensitivities and specificities are summarized in Table 2.

**Table 2:** Prediction of progression by the percentage change in the serum carcinoembryonic antigen (CEA) level at the time of the first response evaluation in patients treated with chemotherapy alone or chemotherapy plus anti-vascular endothelial growth factor (VEGF) or anti-epidermal growth factor receptor (EGFR) monoclonal antibody (mAb).

Progression prediction by CEA	Change in CEA^a^, %	AUC, (95% CI) %	Sensitivity, %	Specificity, %	Negative predictive value, %
Whole cohort	16.5	77 (74.1–81.4)	72.0	70.0	94.2
Chemotherapy alone	16.4	76 (71.7–80.3)	72.5	66.6	92.7
Chemotherapy plus anti-VEGF mAb	− 32.6	74.5 (64.4–84.6)	94.4	48.0	99.2
Chemotherapy plus anti-EGFR mAb	38.9	95.8 (89.1–100)	87.5	94.8	98.9

AUC, area under the curve; mAb, monoclonal antibody.

^a^Change in CEA = (CEA at 1st evaluation—baseline CEA)/baseline CEA.

**Table 3:** Bivariable and multivariable regression analyses of prediction of progression in the whole cohort (n = 1109).

	Bivariable logistic regression			Multivariable logistic regression		
	Unadjusted OR	95% CI	P-value	Adjusted OR	95% CI	P-value
Elevated CEA changes above the cut-off value (*vs* those below the cut-off value^a^)	4.01	2.76–5.81	< 0.001	4.06	2.80–5.90	< 0.001
Age ≥ 60 years (*vs* < 60 years)	0.76	0.52–1.1	0.15	0.71	0.48–1.04	0.08
Primary tumor location, left (*vs* right)	0.85	0.57–1.28	0.44	0.90	0.58–1.38	0.62
BRAF mutant (*vs* wild type)	0.91	0.38–2.16	0.82	0.75	0.30–1.85	0.52

OR, odds ratio; CI, confidence interval.

^a^Cut-off value: CEA increase ≥ 16.5%.

### Correlation between the percentage changes in the CEA level measured at the first response evaluation and clinical outcomes

**Figure 4:**
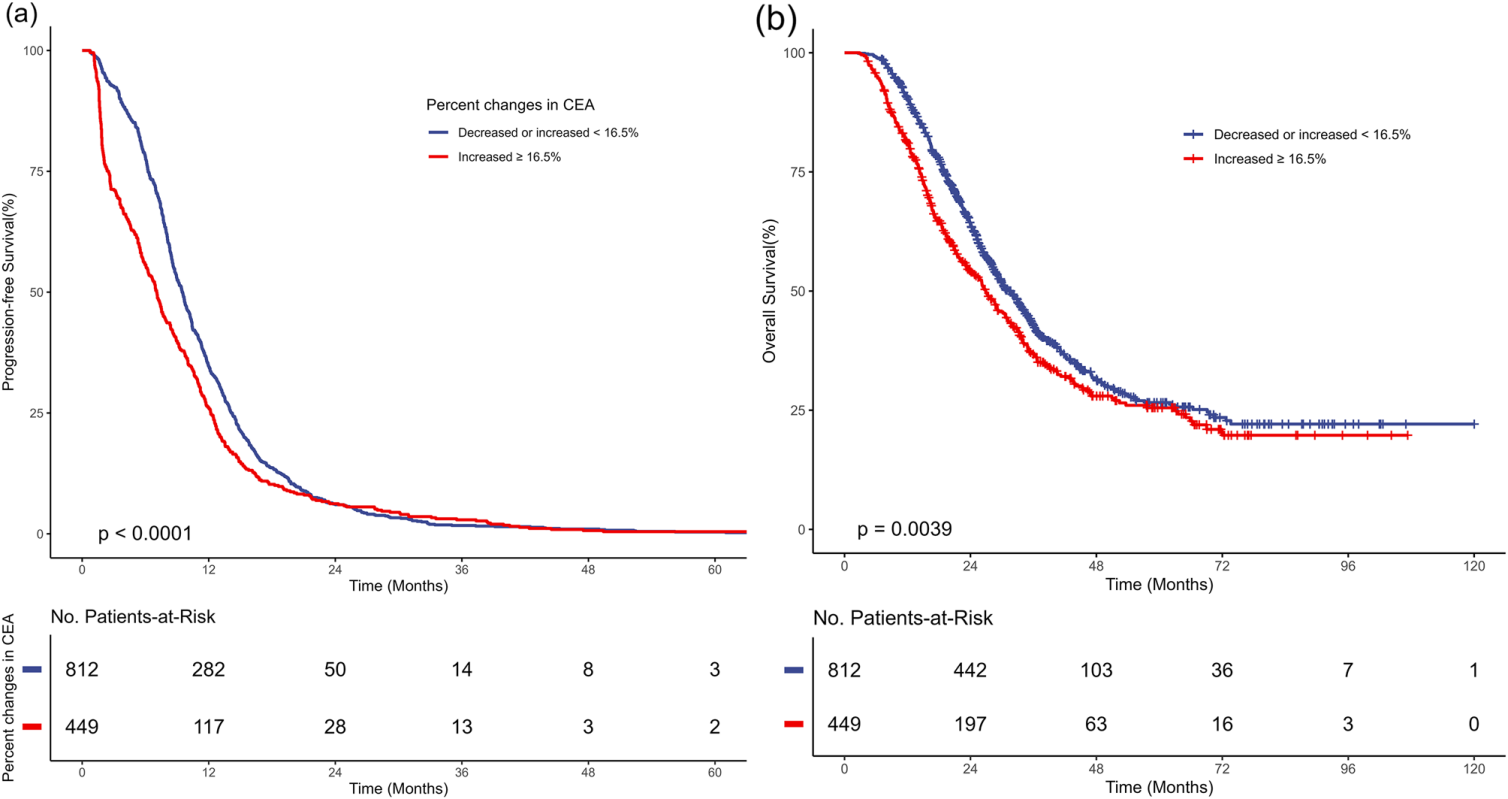
(**a**) Progression-free survival (PFS) and (**b**) overall survival (OS) according to the serum carcinoembryonic antigen (CEA) cut-off value for the whole cohort.

With a median follow-up duration of 43.0 months (95% CI 38.9***–***45.8) among surviving patients, the median PFS and OS in the whole cohort (n = 1261) were 8.71 months (95% CI 8.38***–***9.21) and 29.8 months (95% CI 28.2***–***32.2), respectively. To evaluate the association between the percentage change in the serum CEA level and clinical outcomes in our present mCRC series, we conducted a survival analysis and estimated these variables by stratifying the patients in each treatment arm in accordance with the cut-off value for that group. Among the total study population, 449 and 812 patients were classified as being above (increase in CEA level ≥ 16.5%) and below (decreased or increased < 16.5%) the cut-off value, respectively. The median PFS of the patients in the whole population with changes in CEA level above the cut-off value was 7.07 months (95% CI 6.25***–***7.69), and that for those with changes in CEA level below the cut-off value was 9.5 months (95% CI 8.94***–***9.93, P < 0.0001; Fig. 4a). The median OS was 26.9 months (95% CI 23.4–30.7) and 31.7 months (95% CI 29.1–34.0) for the patients with changes in CEA level above and below this cut-off value, respectively (P = 0.0039; Fig. 4b).

**Figure 5:**
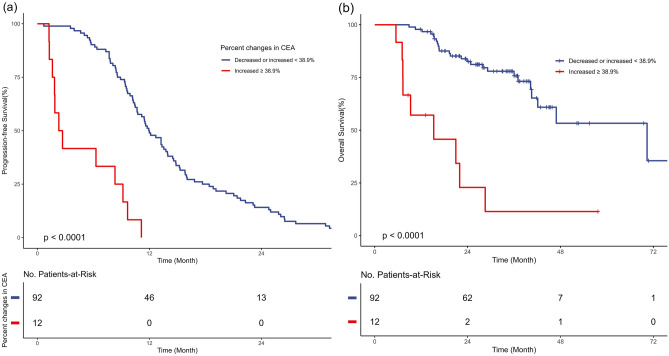
(**a**) Progression-free survival (PFS) and (**b**) overall survival (OS) according to the serum carcinoembryonic antigen (CEA) cut-off value for patients who received chemotherapy plus anti-epidermal growth factor receptor (EGFR) monoclonal antibody (mAb).

Among the patients who had been treated with chemotherapy plus anti-EGFR mAb (n = 104), 12 patients were included in the group with an increase in CEA level above the cut-off value (≥ 38.9% increase), and 92 patients were assigned to the opposite group (a decreased CEA level or an increase < 38.9%). Patients with changes in CEA levels above the-cut-off value who had undergone anti-EGFR mAb combination therapy showed significantly worse PFS and OS outcomes than those with changes in CEA levels below the cut-off value (median PFS, 2.5 months [95% CI 1.84–Not assessed [NA]] vs 12.0 months [95% CI 10.6–14], P < 0.0001, Fig. 5a; median OS, 15.2 months [95% CI 7.29–NA] vs 70.4 months [95% CI 42.1–NA], P < 0.0001, Fig. 5b).

In patients who received chemotherapy plus anti-VEGF mAb, no significant differences were identified between those with changes in CEA above the cut-off value (n = 146) and those with changes in CEA below the cut-off value (n = 120) in terms of the PFS (median PFS, 10.6 months [95% CI 9.44–11.8] vs 11.7 months [95% CI 10.4–13.9], P = 0.11) or OS (median OS, 63 months [95% CI 37.7–NA] vs not reached, P = 0.11) (Supplementary Fig. S2). Among the patients treated with chemotherapy alone (n = 891), those who showed a change in CEA above the cut-off value (n = 353) had a significantly worse PFS than those with a change in CEA below the cut-off value (n = 538) (median PFS, 6.21 months [95% CI 5.4–7.0] vs 8.4 months [95% CI 8.09–8.88], P = 0.00021), but no significant differences were observed in terms of OS between these two groups (median OS, 25.4 months [95% CI 21.2–28.7] vs 27.0 months [95% CI 25.4–29.2], P = 0.35) (Supplementary Fig. S3).

### Prognostic factor analysis

In a multivariable analysis of the total study cohort, patients whose CEA level was increased over the cut-off value (increased by at least 16.5%) had both a significantly worse PFS those with changes in CEA below the cut-off value (HR 1.24, 95% CI 1.08–1.41, P < 0.001) and OS (HR 1.28 [95% CI 1.09***–***1.51], P = 0.003). Furthermore, patients harboring a BRAF mutation showed a significantly shorter PFS (HR 1.41 [95% CI 1.09***–***1.93], P = 0.02) and OS (HR 1.92 [95% CI 1.31***–***2.80], P < 0.001) compared to those without a BRAF mutation. Age ≥ 60 years, tumor grade (moderately or poorly differentiated), and a left tumor location were not associated with OS and PFS in the multivariable analysis (Table 4).

**Table 4:** Univariable and multivariable analyses of prognostic factors in terms of progression-free survival (PFS) and overall survival (OS).

	Progression-free survival							Overall survival					
	Univariable analysis			Multivariable analysis				Univariable analysis			Multivariable analysis		
	HR	95% CI	P-value	HR		95% CI	P value	HR	95% CI	P value	HR	95% CI	P-value
Elevated CEA change above the cut-off value (*vs* below the cut-off value)	1.25	1.10–1.41	< 0.001	1.24		1.09–1.41	< 0.001	1.29	1.10–1.51	< 0.001	1.28	1.09–1.51	0.003
Female (*vs* male)	0.99	0.87–1.11	0.82					1.02	0.87–1.19	0.82			
Age ≥ 60 years (*vs* < 60 years)	1.07	0.94–1.20	0.26	1.04		0.92–1.18	0.45	1.07	0.91–1.25	0.39	1.06	0.90–1.24	0.48
Tumor grade													
Moderately differentiated (*vs* well differentiated)	1.0	0.79–1.25	0.97	0.97		0.77–1.24	0.86	0.88	0.66–1.18	0.4	0.86	0.64–1.14	0.31
Poorly differentiated (*vs* well differentiated)	1.34	1.0–1.81	0.052	1.29		0.95–1.74	0.09	1.58	1.1–2.29	0.014	1.43	0.98–2.08	0.06
Primary tumor location, left (*vs* right)	0.86	0.75–0.99	0.04	0.94		0.81–1.09	0.44	0.77	0.64–0.93	0.006	0.86	0.71–1.04	0.14
BRAF mutant (*vs* wild type)	1.34	1.01–1.78	0.03	1.41		1.03–1.93	0.02	2.05	1.46–2.88	< 0.001	1.92	1.31–2.80	< 0.001
KRAS mutant (*vs* wild type)	1.04	0.92–1.18	0.48					1.06	0.91–1.25	0.44			
NRAS mutant (*vs* wild type)	0.9	0.53–1.51	0.69					1.79	0.55–5.81	0.33			
Deficient MMR (*vs* proficient MMR)	1.26	0.89–1.80	0.2					1.54	1.01–2.34	0.045			
MSI high (*vs* MSS or MSI low)	1.9	1.15–3.45	0.014					1.49	0.74–2.99	0.27			

HR, hazard ratio; CI, confidence interval; MMR, mismatch repair; MSI, microsatellite instability.

## Discussion

The measurement of the serum CEA level as a tumor marker is inexpensive, noninvasive, and convenient, and this underlies why it is a widely used test in patients with mCRC worldwide. In our present study, we observed that CEA changes at the first response evaluation after palliative chemotherapy could predict disease progression with high sensitivity and specificity in a large mCRC cohort. Notably, the sensitivity and specificity of the CEA cut-off value were nearly 90% and 95%, respectively, in patients treated with anti-EGFR mAb plus chemotherapy. In addition, the percentage change in the CEA level was found to be significantly associated with clinical survival outcomes and to be an independent prognostic factor for mCRC.

Several recent reports have indicated that CEA changes can precisely predict non-progression after systemic chemotherapy in mCRC cohorts, which is in line with the findings of our current study^13,14^. Gulahati et al. described a cut-off value for the percentage change in CEA level at the time of the first response evaluation in different treatment groups (chemotherapy alone: − 7.5%, and chemotherapy plus anti-VEGF mAb: − 62.0%) and reported that a change in CEA level was well correlated with long-term clinical outcomes. Morreto et al. proposed that at least a 120% increase in the CEA cut-off value from the nadir was predictive of disease progression after the end of induction chemotherapy in patients treated with the anti-VEGF mAb combination (n = 434). Both studies have suggested that a CT scan could be avoided in approximately 70% of patients and that the measurement of CEA changes was an effective surrogate marker for predicting disease progression. Although the cut-off values for the change in CEA level and the timing of the first response evaluations slightly differed between prior reports, these previous studies and our current data collectively indicate that using CEA changes to predict the disease response in patients with mCRC after systemic therapy has real-world clinical utility, particularly when assessments by imaging studies are inconclusive or unavailable.

Considering the aforementioned studies focused on patients treated with anti-VEGF mAbs or chemotherapy alone, less is known about the role of CEA as a marker of CRC progression in patients who have undergone an anti-EGFR mAb combination regimen in real-world practice. In our present study, among patients without PD at the first response evaluation, we observed that the percentage change in CEA level was significantly lower in patients who had received an anti-EGFR mAb combination (− 63.29%) than in patients who had been treated with an anti-VEGF mAb combination (− 29.06%) (P < 0.001). This result is consistent with those of the pooled analysis in the previous FIRE-3 study, which reported a greater CEA response in patients treated with anti-EGFR mAb than in those treated with anti-VEGF mAb^17^.

Notably, in this regard, the original FIRE-3 study demonstrated that more patients treated with cetuximab experienced an early tumor shrinkage (68%) than those treated with bevacizumab (49%)^18^. Considering that CEA reflects the tumor burden in mCRC^6^, we speculate that an increased CEA response may be a reflection of tumor shrinkage and a greater depth of response (DpR) in patients receiving an anti-EGFR mAb regimen than in those receiving an anti-VEGF mAb treatment. The DpR is a recently proposed efficacy outcome that is defined as the percentage of tumor shrinkage observed at the nadir compared with the baseline^19^, and an increased DpR is regarded as a good prognostic marker for survival outcomes^20–22^. Hence, a greater CEA response may also be a predictive marker of clinical outcomes in patients treated with anti-EGFR mAb, as indicated by our present findings (Fig. 5).

It was of interest that the predictive performance of the CEA response in our current study was lower in the patients who had been treated with anti-VEGF mAb than in those in the other two treatment groups. Similar results were also presented in a previous report, which indicated that the predictive performance of the change in CEA in a chemotherapy alone group was better than that in an anti-VEGF mAb group^13^. Furthermore, we observed no significant differences in the PFS and OS outcomes among patients with mCRC with anti-VEGF mAb according to the CEA-cut-off value for this treatment, in contrast our findings in the anti-EGFR mAb group (Supplementary Fig. S2). These findings suggest that the sensitivity and specificity of using CEA changes to predict mCRC progression and clinical outcomes are less effective in patients treated with anti-VEGF mAb than in those receiving anti-EGFR mAb therapy. One of the potential explanations for this is that CEA may induce angiogenesis independently of VEGF^23,24^. Hence, anti-VEGF mAb may be less effective than anti-EGFR mAb in terms of inducing tumor shrinkage, which was also indicated by the FIRE-3 study^18^. Further investigations will be needed to elucidate the mechanisms behind this.

One strength of our current study was the relatively large sample size. In addition, we evaluated the role of CEA dynamics by comparing different treatment arms including the combined use of biologic agents. Considering that mAb combination regimens are now standard first-line therapies for mCRC, our present findings are highly relevant to current treatment trends for this disease and to real-world clinical practices.

There were several limitations in our current study. First, it was a single-center retrospective study that was susceptible to selection bias. However, our cohort contained a large number of patients treated in a relatively homogenous way with regard to their chemotherapy regimens. Another limitation was that we only evaluated disease response as PD or non-PD and did not further stratify our patients without PD into those with stable disease, a partial response, or complete remission. However, considering that the main purpose of disease evaluation during systemic chemotherapy for mCRC is the detection of PD, our present results may be clinically meaningful in terms of predicting the treatment response. Thirdly, we only evaluated the CEA level measured at the first response evaluation, and the availability of serial follow-up CEA values was limited. Further investigations involving long-term follow-up periods will be needed to evaluate the association between CEA dynamics and disease response in mCRC.

## Conclusion

The measurement of the CEA tumor marker is convenient and noninvasive, and it could be a good surrogate predictive marker of disease progression and survival outcomes in patients with mCRC treated with first-line systemic chemotherapy alone or combination with anti-EGFR mAb, whereas it is less effective in those treated with anti-VEGF mAb. In addition, a greater CEA response may reflect DpR in the anti-EGFR mAb group, which suggests that it could be a potential prognostic marker for these patients. Further investigations including prospective cohort studies are required to definitively evaluate the association between CEA changes and treatment responses in patients with mCRC treated with biologic agents.

## Supplementary Information

Supplementary Information.

## Data Availability

The datasets generated during and/or analyzed during the current study are available from the corresponding author on reasonable request.

## References

[1] Dasari A (2020). ctDNA applications and integration in colorectal cancer: An NCI Colon and Rectal-Anal Task Forces whitepaper. Nat. Rev. Clin. Oncol..

[2] Duffy MJ (2007). Tumour markers in colorectal cancer: European Group on Tumour Markers (EGTM) guidelines for clinical use. Eur. J. Cancer.

[3] Duffy MJ (2014). Tumor markers in colorectal cancer, gastric cancer and gastrointestinal stromal cancers: European group on tumor markers 2014 guidelines update. Int. J. Cancer J. Int. Du Cancer.

[4] Ramphal W (2019). Serum carcinoembryonic antigen to predict recurrence in the follow-up of patients with colorectal cancer. Int. J. Biol. Mark..

[5] Konishi T (2017). Association of preoperative and postoperative serum carcinoembryonic antigen and colon cancer outcome. JAMA Oncol..

[6] Quayle JB (1982). Ability of CEA blood levels to reflect tumour burden: A study in a human xenograft model. Br. J. Cancer.

[7] Hammarström S (1999). The carcinoembryonic antigen (CEA) family: Structures, suggested functions and expression in normal and malignant tissues. Semin. Cancer Biol..

[8] Iwanicki-Caron I (2008). Usefulness of the serum carcinoembryonic antigen kinetic for chemotherapy monitoring in patients with unresectable metastasis of colorectal cancer. J. Clin. Oncol..

[9] Wang WS (2001). Carcinoembryonic antigen in monitoring of response to systemic chemotherapy in patients with metastatic colorectal cancer. Int. J. Colorectal Dis..

[10] Grem JL (1998). The utility of monitoring carcinoembyronic antigen during systemic therapy for advanced colorectal cancer. Oncol. Rep..

[11] Haas RJD (2010). Tumor marker evolution: Comparison with imaging for assessment of response to chemotherapy in patients with colorectal liver metastases. Ann. Surg. Oncol..

[12] Yu P (2018). The dynamic monitoring of CEA in response to chemotherapy and prognosis of mCRC patients. BMC Cancer.

[13] Gulhati P (2020). Threshold change in CEA as a predictor of non-progression to first-line systemic therapy in metastatic colorectal cancer patients with elevated CEA. JNCI.

[14] Moretto R (2021). CEA increase as a marker of disease progression after first-line induction therapy in metastatic colorectal cancer patients. A pooled analysis of TRIBE and TRIBE2 studies. Br. J. Cancer.

[15] National Comprehensive Cancer Network. Colon Cancer https://www.nccn.org/professionals/physician_gls/pdf/colon.pdf (2021).

[16] Van Cutsem E (2016). ESMO consensus guidelines for the management of patients with metastatic colorectal cancer. Ann. Oncol..

[17] Michl M (2016). CEA response is associated with tumor response and survival in patients with KRAS exon 2 wild-type and extended RAS wild-type metastatic colorectal cancer receiving first-line FOLFIRI plus cetuximab or bevacizumab (FIRE-3 trial). Ann. Oncol..

[18] Heinemann V (2021). FOLFIRI plus cetuximab or bevacizumab for advanced colorectal cancer: Final survival and per-protocol analysis of FIRE-3, a randomised clinical trial. Br. J. Cancer.

[19] Mansmann UR (2013). Deepness of response: A quantitative analysis of its impact on post-progression survival time after first-line treatment in patients with mCRC. J. Clin. Oncol..

[20] Cremolini C (2015). Early tumor shrinkage and depth of response predict long-term outcome in metastatic colorectal cancer patients treated with first-line chemotherapy plus bevacizumab: Results from phase III TRIBE trial by the Gruppo Oncologico del Nord Ovest. Ann. Oncol..

[21] Piessevaux H (2013). Use of early tumor shrinkage to predict long-term outcome in metastatic colorectal cancer treated with cetuximab. J. Clin. Oncol..

[22] Schwartzberg LS (2014). PEAK: A randomized, multicenter phase II study of panitumumab plus modified fluorouracil, leucovorin, and oxaliplatin (mFOLFOX6) or bevacizumab plus mFOLFOX6 in patients with previously untreated, unresectable, wild-type KRAS exon 2 metastatic colorectal cancer. J. Clin. Oncol..

[23] Prager GW (2014). Baseline carcinoembryonic antigen (CEA) serum levels predict bevacizumab-based treatment response in metastatic colorectal cancer. Cancer Sci..

[24] Bramswig KH (2013). Soluble carcinoembryonic antigen activates endothelial cells and tumor angiogenesis. Cancer Res..

