# Supplementary material for: CEA dynamics for predicting response after anti-EGFR monoclonal antibody treatment in metastatic colorectal cancer
Source: Sci Rep. 2023 Apr 25;13:6735. doi: 10.1038/s41598-023-33811-x (PMC10130020; doi:10.1038/s41598-023-33811-x)
Supplement: Supplementary file 1 — Supplementary Information. [file 41598_2023_33811_MOESM1_ESM.pdf]

Supplementary Information files

CEA Dynamics for Predicting Response after Anti-EGFR Monoclonal Antibody Treatment in Metastatic Colorectal Cancer

Sora Kang[1,2], Sun Young Kim[1], Yong Sang Hong[1], Tae Won Kim[1], Ki Eun Choi[1], Min Jung Kim[1], Jeong Eun Kim[1]

[1]Department of Medical Oncology, Asan Medical Center, University of Ulsan College of Medicine, Seoul, South Korea

[2]Division of hemato-oncology, Department of Internal Medicine, Chungnam National University Hospital, Daejeon, South Korea.

Correspondence:

Jeong Eun Kim, M.D., Ph.D.

Department of Medical Oncology

Asan Medical Center, University of Ulsan College of Medicine

88, Olympic-ro 43-gil, Songpa-gu, Seoul 05505, South Korea

Tel: +82-2-3010-3945; Fax: +82-2-3010-6961

E-mail: jeongeunkim@amc.seoul.kr

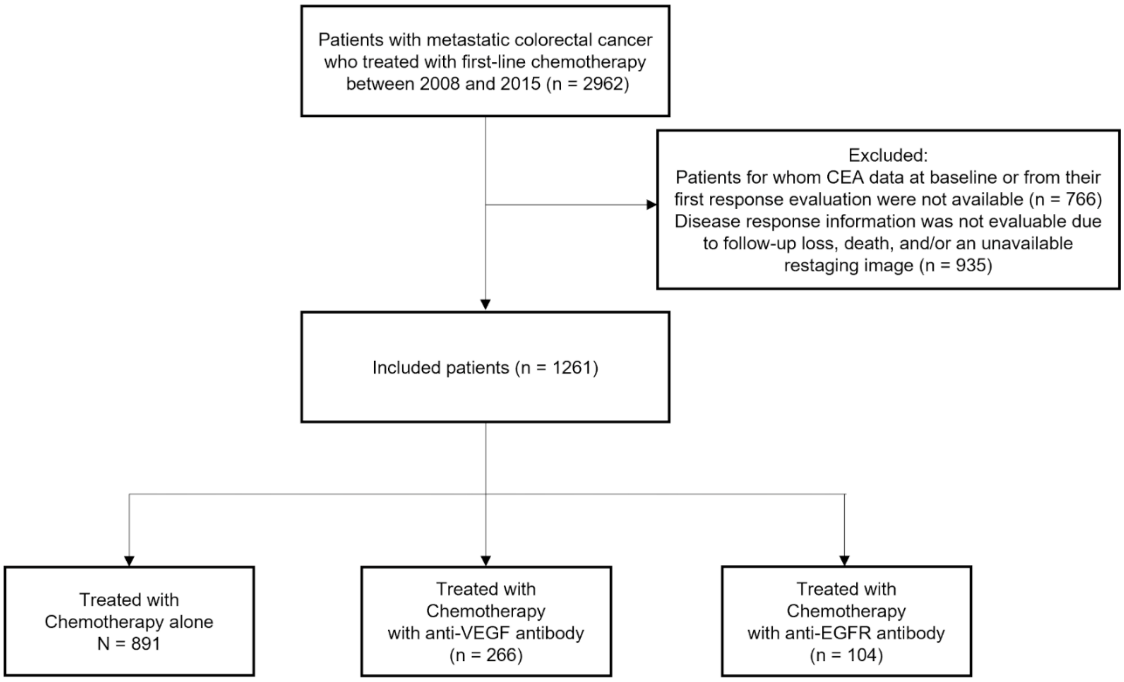

Supplementary Figure S1. CONSORT diagram of the included patients.

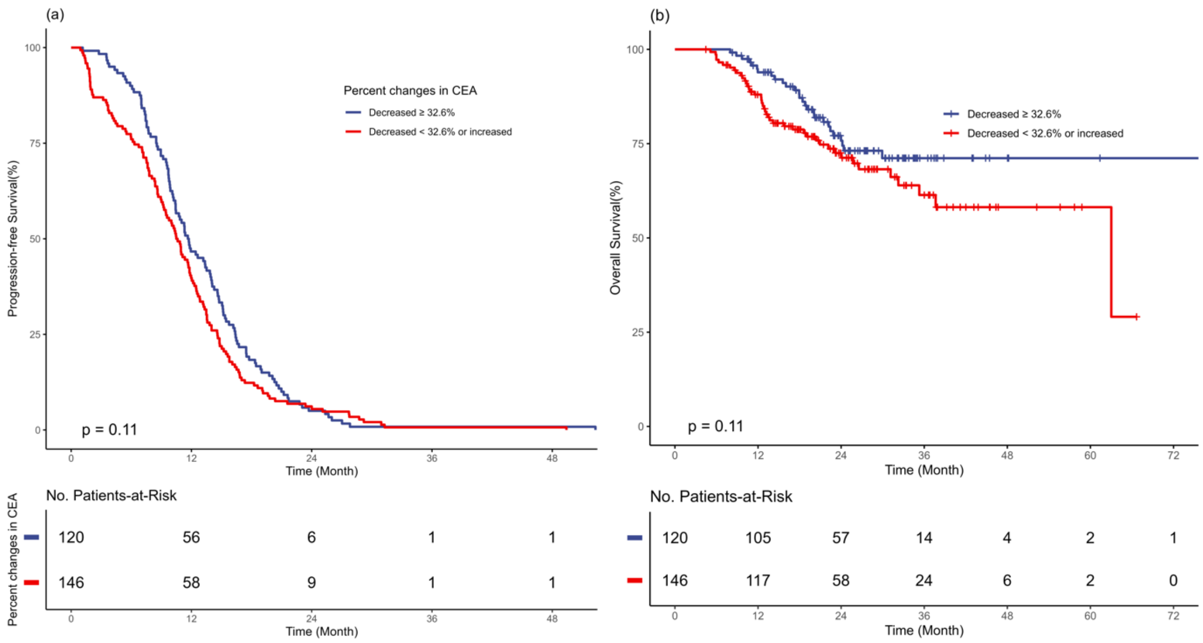

Supplementary figure S2. (a) Progression-free survival (PFS) and (b) overall survival (OS) according to the serum carcinoembryonic antigen (CEA) cut-off value for patients treated with chemotherapy plus anti-vascular endothelial growth factor (VEGF) monoclonal antibody (mAb).

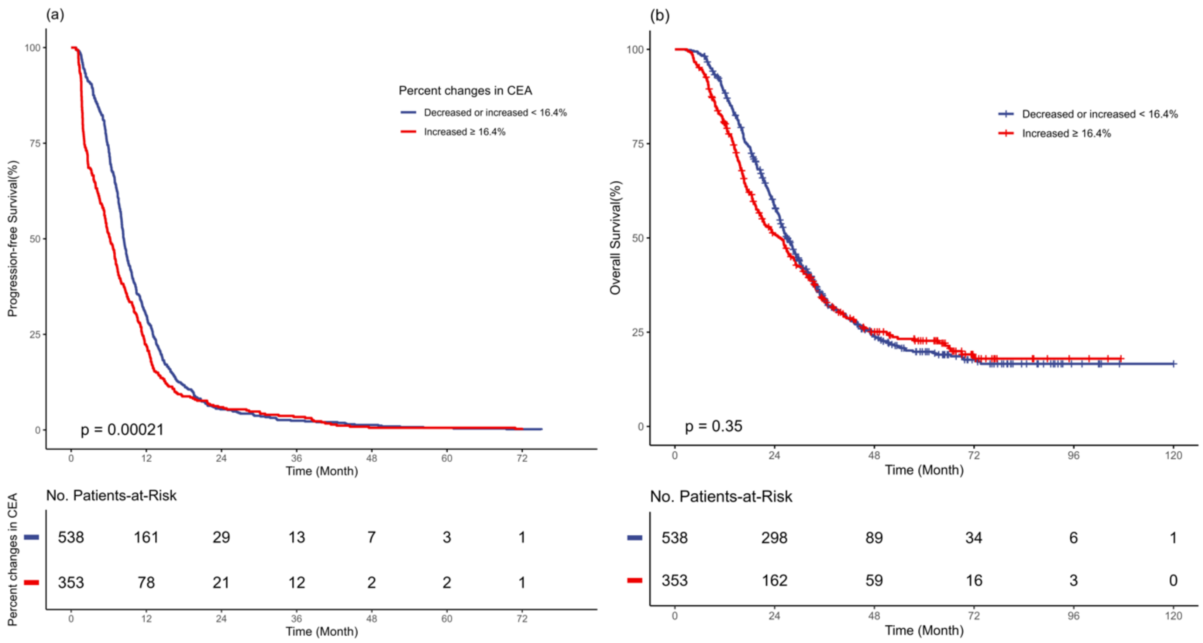

Supplementary figure S3. (a) Progression-free survival (PFS) and (b) overall survival (OS) according to the carcinoembryonic antigen (CEA) cut-off value in patients treated with chemotherapy alone.

Supplementary Table 1. First line chemotherapy regimen in the study patients according to the treatment group

**Chemotherapy alone group**	n = 891 (%)
FOLFIRI	354 (40%)
FOLFOX	305 (34%)
SOX	25 (2.8%)
XELOX	207 (23%)
**Chemotherapy plus anti-VEGF mAb**	n = 266 (%)
Bevacizumab + FOLFIRI	181 (68%)
Bevacizumab + FOLFOX	85 (32%)
**Chemotherapy plus anti-EGFR mAb**	n = 104 (%)
Cetuximab + FOLFIRI	86 (83%)
Cetuximab + FOLFOX	18 (17%)

Abbreviations: mAb, monoclonal antibodies; FOLFIRI, Folinic acid, fluorouracil, and irinotecan; FOLFOX, Folinic acid, fluorouracil, and oxaliplatin; SOX, S-1 and oxaliplatin; XELOX, capecitabine, and oxaliplatin.
